# Case series: raw, whole, plant-based nutrition protocol rapidly reverses symptoms in three women with systemic lupus erythematosus and Sjögren’s syndrome

**DOI:** 10.3389/fnut.2024.1208074

**Published:** 2024-02-27

**Authors:** Brooke Goldner, Kara Livingston Staffier

**Affiliations:** ^1^Goodbye Lupus, Spring, TX, United States; ^2^American College of Lifestyle Medicine, Chesterfield, MO, United States

**Keywords:** systemic lupus erythematosus, Sjögren’s syndrome, raw foods, whole-food, plant-based (WFPB), vegan, disease reversal, autoimmune, lifestyle medicine

## Abstract

Systemic lupus erythematosus (SLE) and Sjögren’s syndrome (SS) are chronic autoimmune diseases. Symptoms of SLE can vary widely but often include fatigue, pain, photosensitivity, and, in some cases, nephritis. SS is frequently characterized by extreme dry eye and mouth, resulting from damage to moisture-producing glands, and is often present in combination with SLE. While the health benefits of plant-based diets have been well-established with respect to weight and cardiometabolic outcomes, less research is available to support the role of diet in treatment and management of autoimmune disease. This case series presents three women with SLE and SS who adopted a nutrition protocol to reverse symptoms of autoimmune disease. The protocol emphasizes leafy greens, cruciferous vegetables, omega-3 polyunsaturated fatty acids, and water, and includes predominately raw foods. The three patients reported dramatic improvements in physical symptoms, with nearly all symptoms of SLE and SS resolving after 4 weeks or less of adhering to the protocol. All three patients have remained symptom-free, two of whom have remained symptom-free for 6+ years with no recent medication use. Patients and practitioners should be made aware of the promising possibility of food as medicine in the treatment of SLE and SS. Future research should explore whether dietary changes may be a potential treatment strategy for individuals suffering from severe symptoms and poor quality of life due to SLE and SS.

## Introduction

Sjögren’s Syndrome (SS) is a chronic, autoimmune disorder characterized by damage to moisture-producing glands, resulting in dry eyes (xerophthalmia) and mouth (xerostomia). It can also encompass other symptoms including joint pain, swelling, rashes, and fatigue. SS may be present in combination with other autoimmune diseases, such as systemic lupus erythematosus (SLE). SLE is also a chronic, autoimmune disease, but symptoms can vary widely in both scope and severity. Common symptoms include extreme fatigue, joint pain and swelling, hair loss, photosensitivity, and a butterfly-shaped rash across the cheeks and nose. More serious damage such as nephritis and pleuritis can also occur. The prevalence of SLE is estimated to be 43.7 (range 15.87 to 108.92) per 100,0000 persons globally, with a prevalence of 78.73 (28.61 to 196.33) per 100,000 in women and 9.26 (3.36 to 22.97) per 100,000 in men (1). Estimates of the prevalence of SS in SLE patients in the literature vary from 6–19%, likely due to the use of different criteria to define SS (2–4).

There is an extensive amount of research documenting the health benefits of plant-based diets with respect to weight and cardiometabolic outcomes (5–11). A growing body of research has shown that lifestyle change, specifically adherence to more plant-based diets, may both protect against autoimmune disease as well as improve symptoms of autoimmune dysfunction (12, 13). A 2022 study suggested that individuals adhering to a vegetarian diet had lower odds of being diagnosed with SLE compared to non-vegetarians (14). This case series details three women with SLE and SS who reported remission of symptoms after following a strict, customized, plant-based nutrition protocol for recovery (recovery protocol) that eliminates all processed foods and was developed by the author, Dr. Goldner (BG). The three cases presented completed the author’s Rapid Recovery Program (RRP), in which they work with the author (BG) on a daily basis for personalized recommendations and maximum support with respect to adhering to the recovery protocol. The RRP is offered as either a 4-week, one-on-one program or a 6-week group program.

Whole-food, plant-based (WFPB) diets eliminate processed foods, added oils, sugars, and animal products, and the recovery protocol shares similarities with a WFPB diet; however, it is further refined to focus on predominately raw foods and high intakes of leafy greens and cruciferous vegetables, omega-3 polyunsaturated fatty acids (whole, ground flax or chia seeds; cold pressed flaxseed oil), and water. On the recovery protocol, while raw vegetable intake is allowed *ad libitum*, minimum daily intakes are set as follows: 16 oz. leafy greens (i.e. spinach, kale) and cruciferous vegetables; ½ cup flax or chia seed or 3 tablespoons cold pressed flaxseed oil; and 96–128 oz. of water. Fruit is recommended at no greater than 25% of total dietary intake to ensure that patients are able to consume the recommended amount of raw vegetables before reaching satiety. Vitamin B12 and vitamin D supplementation is recommended. If remission of symptoms is achieved, patients are allowed to step down to a maintenance phase which allows for the incorporation of some cooked whole, plant foods as well as more fruits, and the addition of nuts and seeds. Patients are advised to try one new change at a time, waiting 3–5 days in between to ensure there is no re-emergence of symptoms. If symptoms emerge, patients return to the recovery protocol. The maintenance phase remains 100% plant foods with 75% of intake recommended to be raw, continuing with daily smoothies or salads, and incorporating 64 oz. of water or more unless otherwise recommended by the patient’s physician. Fruit is not restricted, and other whole, plant foods such as legumes and whole grains may be incorporated during maintenance. After 6 months of remission, processed vegan foods, foods with sugar or oil, and alcohol are allowed 1–2 times/week (termed ‘recreational eating’), while otherwise continuing the maintenance protocol, if patients remain asymptomatic (Table 1).

**Table 1:** Summary of dietary recommendations during rapid recovery and maintenance phases.

	Initial rapid recovery/reversal phase	Maintenance^5^
Include	Raw vegetables (unlimited); focus on high intake of leafy greens and cruciferous vegetables, minimum recommended 16 oz. per day^1^ Fruits (recommended ≤25% total dietary intake/day) Whole, ground flax and chia seeds (minimum 1/2 cup recommended per day initially and increase as tolerated)^2^ or 3 tbsp. cold pressed flaxseed oil Water^3^ Vitamin B12 and D supplementation 1 tsp. salt/day *Ezekiel bread (not typically included) allowed for Case 1, due to increased hunger during pregnancy*	Vegetables (focus on high intake of cruciferous vegetables); recommended 75% raw Fruits (no recommended restriction) Seeds and nuts Whole, ground flax and chia seeds (minimum 1/4 cup/day) Water, recommended 64 oz. or more Intact, whole grains Legumes Vitamin B12 and D supplementation 1 tsp. salt/day
Eliminate	All animal products (i.e., meat, dairy, seafood) Added oils^4^ Processed foods Added sugars Cooked foods Grains Legumes	All animal products (i.e., meat, dairy, seafood) Added oils^4^ Processed foods, added sugars, alcohol (exception noted below) After 6 months of remission, processed vegan foods, foods with sugar or oil, and alcohol are allowed 1-2 times/week (termed ‘recreational eating’), while otherwise continuing the step-down^5^ or maintenance protocol. This is only allowed when individuals remain asymptomatic.

1 Recommend at least 64 oz green smoothie per day as a way to more easily consume the recommended vegetables.

2 Assess gastrointestinal discomfort; recommended to spread intake throughout the day.

3 If body weight ≥ 120 lbs., recommended 96–128 oz. per day (approx. 1 gallon); if body weight < 120 lbs., recommended water is oz. of body weight (i.e., 80 lbs. = 80 oz. water/day); some patients (i.e., Case 3) may need to individually adjust requirements with their physician.

4 Exception for cold pressed flaxseed oil.

5 When moving from initial rapid recovery phase to maintenance phase, patients are advised to try one new change at a time, waiting 3–5 days in between to ensure there is no re-emergence of symptoms. If symptoms emerge, patients return to the recovery protocol.

## Case descriptions

### Case 1

Case 1, a 40-year-old female, was approximately 9 months pregnant when diagnosed with SLE and SS in 2013 and reported experiencing symptoms since at least 2010. She was taking hydroxychloroquine for management of SLE and SS and aspirin to reduce risk of blood clot and miscarriage. She experienced extreme photosensitivity, fatigue, and pain in the legs that required her to spend much of the day lying down. She also experienced extreme dry skin, including cracks that would not heal, dry eye, and dry mouth. Other symptoms included stomach cramping, diarrhea, and severe pelvic pain. Interested in natural treatments, she contacted the author (BG) in April 2017 and immediately began her 4-week, one-on-one RRP to which she reports being 100% adherent. She notes that while she did consult with her rheumatologist prior to beginning the RRP, she did not consult with her obstetrician because, in her words “I was desperate to get healthy and scared of a flare-up after delivery, which is what happened with my first pregnancy.” She consumed one 64 oz. green smoothie daily, composed predominately of raw leafy greens such as kale or spinach, water, and fruit added for flavor. She also incorporated flaxseed, first starting with ½ cup and working her way to a full cup. Her other meals throughout the day included foods such as salads and raw vegetables, in addition to drinking a gallon of water. The only non-raw food that she consumed was Ezekiel bread, a sprouted whole grain bread, at the recommendation of the author, which is not typically included with the recovery protocol but was allowed due to increased hunger during pregnancy.

Two days into the RRP, she reported that her pelvic pain had stopped. Most other symptoms completely resolved over the 4 weeks, including the dryness, pain, and fatigue. The cracks in her skin were actively healing, and completely resolved within 3 months of beginning the RRP. She remained active during pregnancy, gave birth to a healthy child, recovered quickly from her C-section, and continued with no pain and increased energy post-birth.

**Figure 1:**
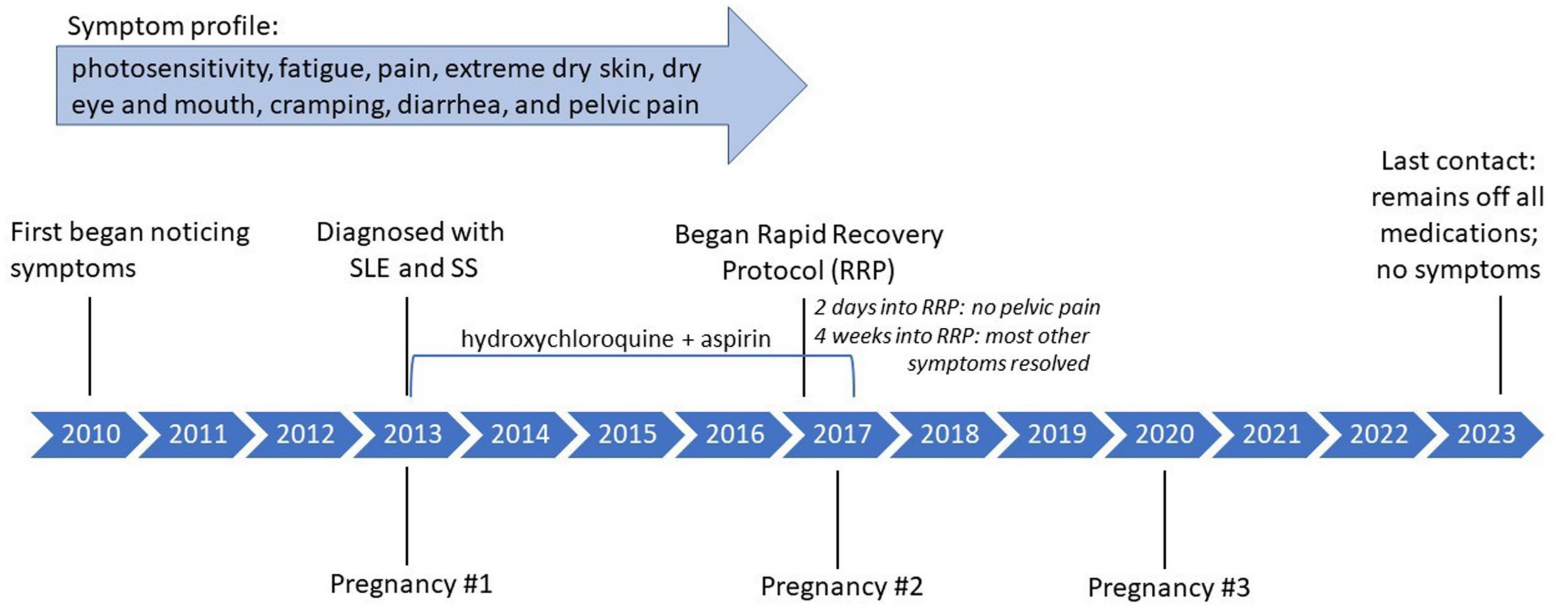
Case 1: timeline of symptoms and medication use.

Two months after completing the RRP she went outside for the first time and experienced no photosensitivity. By approximately 6 months after completing the RRP, she had discontinued both hydroxychloroquine and aspirin. Post RRP, her lab results showed a decrease in SS-B, a marker antibody for SS, as well as a decrease in partial thromboplastin time (PTT), for which a prolonged PTT (>40) may suggest SLE (Table 2). She transitioned to a maintenance diet, still including no processed foods, 40 oz. per day of green smoothie, a gallon of water, and approximately 90% raw, whole plant foods during the day with a cooked plant-based meal for dinner. As of last contact (December 2023), she remained off all medications, had an additional healthy pregnancy and delivery in 2020, and reported no recurrence of symptoms. She reports feeling that the protocol “was such an easy fix for my illnesses.” Please see Figure 1 for a timeline of symptoms and treatment. The Supplementary File includes a personal testimony from the case.

**Table 2:** Case 1: summary of laboratory testing from 2014 to 2018.

	Date of laboratory testing						
	6/23/14	8/4/16	10/20/16	1/6/17	6/19/17	10/19/17	3/8/18
DNA (DS) antibody, IU/ml^a^	3 (negative)	5 (indeterminate)	5 (indeterminate)	6 (indeterminate)	–	–	–
Sjögren’s antibody: SS-A	>8 (positive)	–	–	–	>8 (positive)	–	>8 (positive)
Sjögren’s antibody: SS-B	3.3 (positive)	–	–	–	<1 (negative)	–	<1 (negative)
ANA^b^	positive	–	–	–	–	positive	–
Lupus Anticoagulant (LAC)^c^	–	–	–	–	–	not detected	–
Cardiolipin antibody-IgM^d^	25 (high)	24 (high)	–	–	–	–	–
Cardiolipin antibody-IgG^d^	<14 (negative)	<14 (negative)	–	–	–	–	–
Cardiolipin antibody-IgA^d^	–	<11 (negative)	–	–	–	–	–
PTT screen (LAC)^e^	43 (high)	–	–	–	–	37 (normal)	–
CRP (mg/dL)^f^	0.1 (in range)	–	–	–	–	–	–
C3 (mg/dL)^g^	96 (in range)	–	–	–	–	91.6 (in range)	–
C4 (mg/dL)^g^	38 (in range)	33 (in range)	–	–	–	28.5 (in range)	–

a Antinuclear antibody frequently present in individuals with SLE; may suggest more serious SLE.

b identifies autoantibodies characteristic of autoimmune disorders; most individuals with SLE have positive ANA.

c Lupus anticoagulants (LA) are antiphospholipid antibodies that specifically target phospholipids and associated proteins and can interfere with blood clotting.

d Cardiolipin is another antiphospholipid antibody. The presence of LA and cardiolipin do not indicate a diagnosis of SLE; however, these tests can indicate potential risk of complications from antiphospholipid antibodies in SLE patients, such as miscarriage, blood clot, or stroke.

e Partial thromboplastin time (PTT) measures time it takes for blood to clot and can identify if blood contains anticoagulant antibodies.

f Blood marker for inflammation.

g Low levels of complement C3 and C4 reflect inflammation.

– indicates test not performed.

### Case 2

Case 2, a 54-year-old female, experienced photosensitivity, butterfly rash, itchy scalp, and constant fatigue since approximately 2006. In subsequent years, she was in and out of the hospital with pleurisy, as well as joint stiffness in the fingers, elbows, and knees. She experienced severe dry mouth, which interfered with eating, as well as severe dry eye that began around May 2015. She had been diagnosed with SLE based on bloodwork on July 5, 2015 and was prescribed hydroxychloroquine which she reports did not improve symptoms. Months later, she was diagnosed with SS. In February 2016, she saw a neurologist due to neuropathy (fingers, arm) who recommended she reach out to the author (BG) which she did on February 14, 2016.

Case 2 read the author’s book (BG) detailing the recovery protocol (15) and eliminated all meats, sugars, processed foods, and added oils except for cold pressed flaxseed oil from her diet and began to incorporate more greens. She had an initial consultation with the author (BG) about 2 weeks later and further refined her diet to incorporate a higher amount of greens, such as kale and spinach, which she estimates at approximately 11 cups per day (range 2.5–16), as well as raw fruits and omega 3 (chia seeds/flaxseed oil). She enrolled in the 4-week RRP and, after further consultation, her diet included two 32 oz. green smoothies per day, a salad at lunch, and dinner consisting of foods such as kale, cabbage, and Brussels sprouts.

Her symptoms, including neuropathy, joint stiffness, pain, fatigue, itchy scalp, and photosensitivity, resolved within 14 days, and dry eye improved over several months. After 6 months, her ophthalmologist confirmed in an exam that she no longer had any visible eye inflammation and showed no physical symptoms of dry eye. Her anti-double stranded DNA (anti-dsDNA) test results, for which higher numbers suggest higher presence of SLE antibodies, decreased from 20 IU/mL in January 2016 to 16 IU/mL in January 2017. Although 16 is still above normal, it should be noted that dsDNA can remain positive even when clinical symptoms are not present.

**Figure 2:**
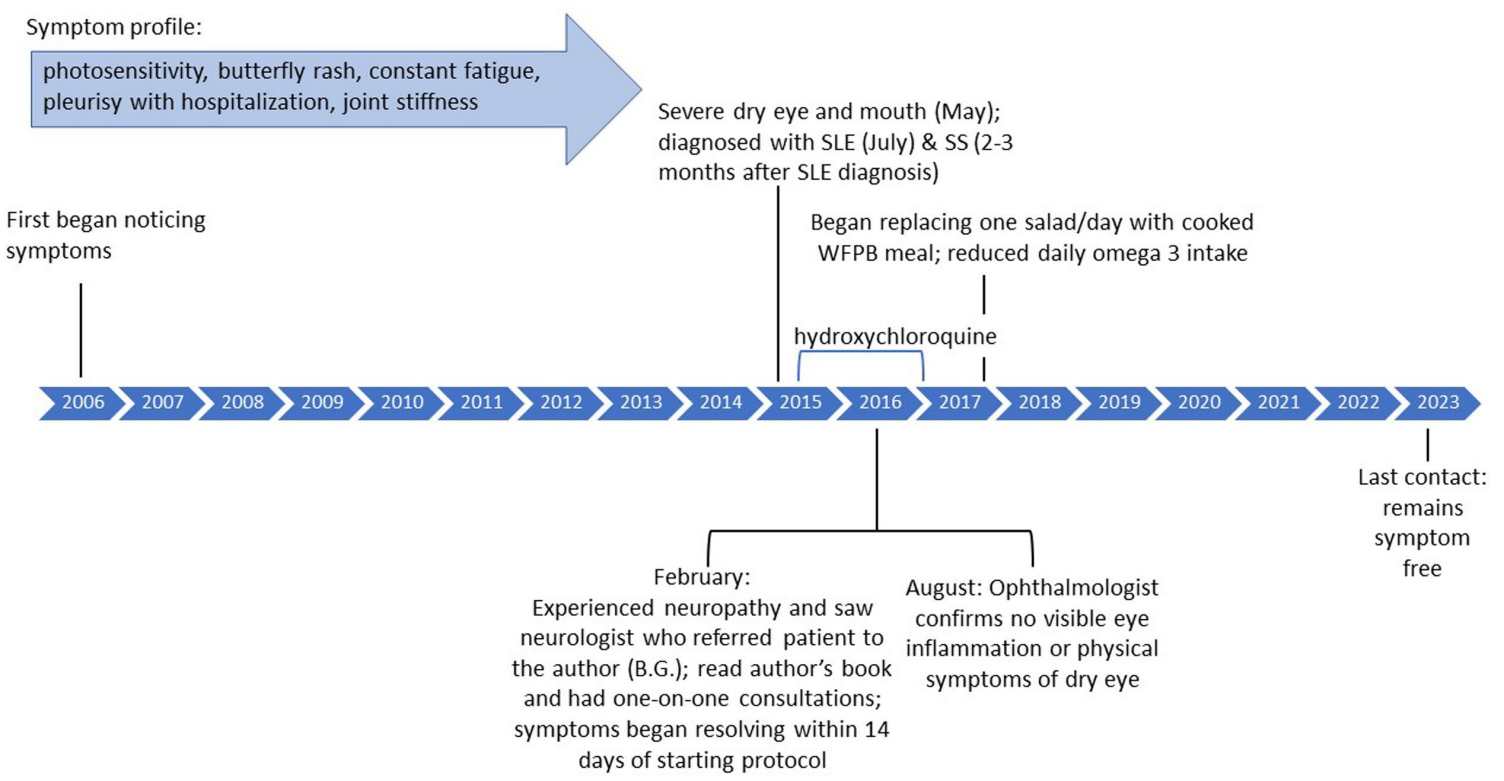
Case 2: timeline of symptoms and medication use.

She maintained the diet as prescribed by the author (BG) for a year, at which point she started to replace one salad with a cooked plant-based meal, still eliminating all processed foods. She discontinued hydroxychloroquine in January 2017. She reports eating a vegan cake at her wedding made with processed sugar and experiencing joint stiffness after. However, when she went on a cruise and ate unprocessed, whole plant-based foods, she did not experience any symptom flare-ups. After approximately 2 years, she significantly reduced the amount of omega 3 consumed but has not experienced any flares in symptoms or changes in blood work. As of last contact in July 2023, she remains symptom free and continues to eat a whole-food, plant-based diet that incorporates both daily green smoothies as well as cooked foods. She stated “I am doing so well….I’m convinced that it [the recovery protocol] saved my life.” Please see Figure 2 for a timeline of symptoms and treatment. The Supplementary File includes a personal testimony from the case.

### Case 3

Case 3, a 45-year-old female, reports that from approximately 2003–2008, she often felt sick and achy with flu-like symptoms, migraines, intermittent dizziness, weakness, and increased feelings of dry mouth. She reports having visited her primary care physician (PCP) on multiple occasions, who was unable to determine a cause of her symptoms, although she was never tested for autoimmune disease. In 2008, after the birth of her fourth child, she was referred to a rheumatologist who was also unable to determine a diagnosis. She continued seeing her PCP for her symptoms until he later, still unable to diagnose her, referred her to a psychiatrist. At this time, blood work detected anticardiolipin antibodies, which remained present for the next 7–10 years. Her child was then diagnosed with neonatal lupus at around 10 weeks old. This prompted testing of Case 3 and a subsequent diagnosis of SS based on the presence of antibodies (anti-SS-A and anti-SS-B) and dry eye symptoms. Antinuclear antibodies (ANA) were also detected; however, SLE was not officially diagnosed due to lack of typical clinical symptoms. Of note, the child no longer tested positive for antibodies after around 1 year of age and currently remains antibody free at 15 years of age. Around 2010, Case 3 began taking levothyroxine (100 mg/day) as she was found to have high antibodies for Hashimoto’s disease, and her parents both had experienced thyroid disease. She was also prescribed an immunosuppressant (azathioprine) in 2010 that did not improve symptoms and she eventually stopped, and was then prescribed methotrexate. In 2012, she began hydroxychloroquine which did help to manage symptoms.

For the next decade, she continued to experience the symptoms previously described. Around 2014, she sought care with a rheumatologist and was diagnosed with a ‘non-specific’ autoimmune condition because she did not have typical SLE symptoms. In 2017, she was officially diagnosed with SLE based on the presence of antibodies and increasing symptoms such as constantly feeling sick and revolving nerve pain in 6–8 inch wide areas of her skin.

**Figure 3:**
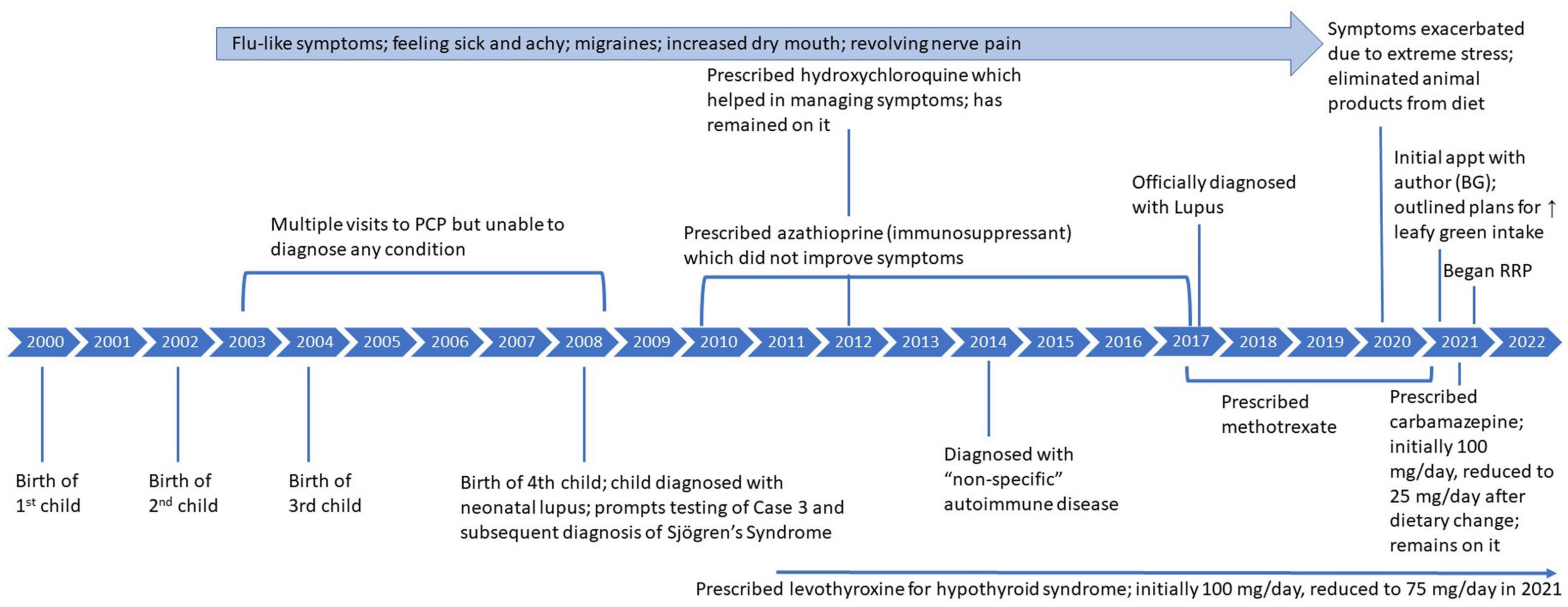
Case 3: timeline of symptoms and medication use.

In September 2020, due to extreme stress related to Covid-19, her symptoms exacerbated significantly, including extreme fatigue, pain, a migraine that lasted for 4 months, a diagnosis of hyponatremia with hospitalization, and extreme light sensitivity and eye pain, described as varying from a feeling of “someone squeezing my eyes” to a feeling of grittiness or sand in the eyes that prevented her from being able to open both eyes at the same time. Please see Figure 3 for detailed timeline of symptoms and treatment.

She reports having followed a keto diet ‘off and on’ from 2007 to 2020 but had not experienced any improvements in symptoms. After the extreme exacerbation of symptoms in 2020, she read the author’s (BG) book detailing the recovery protocol (15) and started to follow a plant-based diet with no animal products, specifically incorporating spinach (approximately 10 oz./day) but not restricting sugar. She noticed small improvements in symptoms. She began working with the author (BG) in February 2021, at which point it was emphasized that she needed to greatly increase intake of greens and cruciferous vegetables and eliminate added sugar. Initially, although “not super strict” about adhering to the plan outlined by the author (BG), she saw fatigue and energy improve in just 2 weeks, migraines improved in about 1 month, and dizziness subsided later. At this time (2021), her physician also prescribed carbamazepine to help with trigeminal neuralgia. When she was diagnosed with Covid-19 in 2021, her doctor suggested temporarily stopping methotrexate, and she never restarted as symptoms were significantly improving.

In June 2021, motivated to further improve symptoms, she completed the author’s (BG) 6-week RRP. Within 3 weeks of strict adherence to the RRP, her migraines and revolving pain on her skin resolved, and her dry mouth and dry eye significantly improved and continued to improve with continued adherence to the protocol. Her recovery diet was comprised predominately of large salads, and she estimates that she consumed approximately 1.5–2 lbs. of raw, cruciferous vegetables and/or spinach, approximately 1 pound of other vegetables, microgreens, 6 tbsp of flaxseed oil and, initially, approximately 128 ounces of water per day. In November 2022, she was diagnosed with hyponatremia by her regular physician, although was not hospitalized as she was in 2020. It should be noted that her endocrinologist previously diagnosed her with a pituitary tumor that may be contributing to syndrome of inappropriate antidiuretic hormone secretion (SIADH), thus resulting in hyponatremia. Under the care of her medical team, she slowly reduced her water intake to 96 oz. and, finally, 60–80 oz.

The key laboratory results of interest to her physician post-recovery were complement protein 3 (C3), complement protein 4 (C4), white blood cell count, red blood cell count, and ANA (Table 3). While ANA remained positive pre- and post-recovery, C3, C4, and white blood cell counts improved, moving into normal ranges. A few months after symptoms resolved, she tried to introduce additional plant-based foods such as beans, more fruit, and almond flour into her diet but reports not feeling as well and returning back to strict adherence to the recovery protocol. After 8–12 months adhering to the protocol and eating mostly raw foods, all symptoms had resolved. She has felt symptom free since early spring 2022.

**Table 3:** Case 3: key laboratory results pre- and post-rapid recovery protocol.

	September 2020 *Pre RRP*	May 2022 *Post RRP*	March 2023
C3 (mg/dL)^1^ *Normal range 83–180*	53	84	104
C4 (mg/dL)^1^ *Normal range ≥ 10.0*	<8.0	15.2	20.2
White blood cell count (K/uL) *Normal range 3.7–11.1*	1.9	3.7	4.9
Red blood cell count (M/uL) *Normal range 3.60–5.70*	3.50	3.23	4.16
ANA *Normal reading: Negative*	Positive	Positive	Positive

1 Low levels of complement C3 and C4 reflect inflammation.

As of last contact (December 2023), she has remained adherent to a predominately raw, plant-based diet with no processed foods, incorporating a small amount of cooked vegetables (approximately 6% of her diet), some fruit and beans, and consuming 2 tbsp/day of cold pressed flaxseed oil or chia seed oil. She is currently on hydroxychloroquine, levothyroxine, and carbamazepine, although her doses of the latter two have been decreased since beginning the recovery protocol. She remains under the care of her medical team, monitoring for hyponatremia, and currently consumes no more than 25–35 oz. of water daily. She reports feeling healthy, being active, and having dramatically improved quality of life.

Please see Figure 3 for a summary of initial symptoms and resolution across all three cases. The Supplementary File includes a personal testimony from the case. Please see Table 4 for a summary of improvement in symptoms for all 3 cases following the recovery diet.

**Table 4:** Summary of symptom improvement among three cases.

	Symptoms prior to recovery protocol	Symptoms resolved immediately (≤1 month) after start of RRP	Symptoms resolved later (>1 month)
Case 1	Extreme photosensitivity, fatigue, pain in the legs, extreme dry skin, dry eye, dry mouth, stomach cramping, diarrhea, severe pelvic pain	Pelvic pain resolved (2 days); dry eye, dry mouth, pain in the legs, stomach cramping, diarrhea, fatigue (4 weeks)	Cracks in skin (3 months); photosensitivity (2 months)
Case 2	Photosensitivity; butterfly rash; constant fatigue; joint stiffness in the fingers, elbows, and knees; severe dry mouth and dry eye; eye inflammation; neuropathy	Neuropathy, joint stiffness, fatigue, and photosensitivity; butterfly rash; dry mouth (2 weeks)	Eye inflammation and dry eye (6 months)
Case 3	Flu-like symptoms; migraines; intermittent dizziness; weakness; dry mouth; revolving nerve pain in skin; extreme fatigue; extreme light sensitivity; eye pain; trigeminal neuralgia.	Migraines, revolving pain on skin, dry mouth, dry eye (3 weeks)	Flu-like symptoms; dizziness; weakness; dry mouth, dry eye, fatigue, light sensitivity, eye pain, trigeminal neuralgia (8–12 months)

## Discussion

This case series details three women, 40, 54, and 45 years of age, with SLE and SS who reported debilitating symptoms that severely compromised both physical health and quality of life. They self-reported remission of symptoms beginning a month after adhering to a customized nutrition protocol for recovery, consisting of whole, predominately raw, plant foods. While a case series cannot establish causality, these results show dietary change as a potentially promising approach for treatment of SLE and SS. At the time of writing, Cases 1 and 2 reported having remained adherent to the maintenance protocol and have remained symptom free with no medication use for 6 and 7 years, respectively, showing the potential for long-term symptom remission. Other case reports in the literature similarly support the use of plant-based diets in reversing diseases such as chronic kidney disease and outcomes related to cardiovascular disease (16–18), suggesting that the recovery protocol may be successful for a number of other chronic diseases.

While this case series details a specific nutrition protocol for recovery, previous research has supported the potential for diet to improve SLE-related symptoms (19–22). Constantin et al. discusses the importance of personalized diet in maintaining homeostasis, remission, and physical well-being for SLE patients (21). A 2020 review by Islam et al. suggests that low-calorie, low-protein diets high in fiber, PUFA, vitamins, minerals, and polyphenols may provide a macronutrient and micronutrient profile that benefits SLE patients (23). SLE and SS are chronic inflammatory diseases (2, 24), and it is well-established that diets high in fruits and vegetables can reduce both inflammation and oxidative stress. The recovery protocol documented in this case series is high in antioxidant-rich fruits and vegetables which may help to reduce the oxidative stress that is characteristic of SLE and can contribute to more severe SLE symptoms (20, 24–26), and diets higher in fruits and vegetables may reduce proinflammatory mediators (27).

The recovery protocol documented in this case series is high in omega 3, a polyunsaturated fatty acid (PUFA) shown to reduce inflammation. One study in autoimmune, Lupus-prone mice suggested that fish oil (omega 3) inhibited production of pro-inflammatory cytokines and improved kidney injury (28), and omega-3 intake has been linked to improved outcomes in SLE patients, including reduced inflammation, disease activity, and oxidative stress (22). Other published work has shown that supplementation, such as with omega-3, can help reduce SLE-related sequelae (22, 29) and disease activity (30, 31) and that omega 3 may reduce the production of inflammatory markers, cytokines and CRP to improve inflammation (32–34). Finally, PUFA supplements, in particular omega-3, may be an effective treatment for non-specific dry eye disease (35, 36). Various mechanisms have been hypothesized to theorize why omega 3 fatty acids may improve outcomes associated with SLE, including by reducing disease activity, reducing inflammation, regulating adipokine production, and improving endothelial function (37). The use of high doses of omega-3 rich foods and oils as part of the recovery protocol is a novel test of this previous research in a clinical setting. However, further research is needed to improve understanding of mechanisms and also to further explore whether different types of omega 3 fatty acids may exert different effects (ALA vs. EPA/DHA) and the relative contributions of the omega-3 fats vs. other aspects of the recovery diet.

SLE is a heterogenous, multi-system disease that is often difficult to diagnose and complex to treat (38), as evidenced by the extensive period that Case 3 experienced illness before a formal diagnosis of SLE was made. SLE disease activity is associated with negative physical and mental health outcomes, including organ damage and poor quality of life (39, 40). Despite the fact that life expectancy is generally favorable (41, 42), racial and ethnic minority groups experience more severe outcomes (43), and SLE can dramatically affect all areas of a patient’s life, including physical health, mental health, and social relationships (44). Recent research has highlighted poor physical and mental health related quality of life in patients with SLE, with literature indicating that SLE can cause pain and fatigue (45, 46), poor sleep quality (47), feelings of fear, anguish, and social exclusion (48), decreased sexual function (49), concerns around fertility and reproductive health in women (50), and challenges around economic costs, status, and employment (51). In addition, quality of life may be affected differently at different phases of disease (52). Severe SLE flares were found to be associated with reduced health-related quality of life (53). As described, all three cases experienced a wide array of symptoms and significant negative impacts on physical health, mental health, and quality of life; significant improvements in all symptoms were observed after following the prescribed recovery protocol. It is notable that significant improvement in all symptoms, improvements that were superior to that achieved with medication, were observed.

Although many pharmacological therapies for SLE exist, there is potential for both short- and long-term side effects (54–56), and a recent observational study showed that some pharmacological treatments yield better improvements in quality of life than others (57). Pharmacological therapies are typically targeted at reducing disease activity and preventing mortality; remission is not always possible (58). The 2021 Definitions of Remission in SLE (DORIS) International Task Force highlighted remission as an “aspirational goal in clinical care” and defined remission as minimal to no disease activity based on two indices [Systemic Lupus Erythematosus Disease Activity Index (SLEDAI) and Physician Global Assessment], irrespective of serology, and allowing for use of some pharmaceuticals including antimalarials (59). Although this case series was not able to utilize a disease index such as SLEDAI to assess symptoms, the three cases self-reported no symptomatic disease activity in the period following the adoption of the recovery protocol. Two of the cases were able to stop use of all pharmacological treatment, and all three cases had medications deprescribed under the care of and with monitoring by their physician, not the authors. Despite the experience of these three patients, it is important to note that pharmacological treatment, particularly with hydroxychloroquine (HCQ), remains the current standard of care for SLE (60), and adequately dosed HCQ has been shown to be largely well tolerated, is associated with lower risk of disease activity, and has a protective effect on survival (61, 62). Thus, while this case series is promising, further research such as randomized controlled trials would be needed to examine the role of diet and improved outcomes before broad recommendations for deprescribing of SLE medications could be considered. Although not examined in this case series, remission of symptoms would likely also result in reduced healthcare costs to both patients and payors.

It is important to note the limitations of this work. Case series are observational, descriptive studies and do not utilize a control group. In the hierarchy of evidence, case series provide weak evidence for causality on their own and best serve to provoke consideration for future research. Case series are, however, important for sharing experiences between clinicians and generating hypotheses that may lead to future research (63). As this is a case series, we cannot conclude whether the dietary change alone was responsible for the observed improvement in symptoms; it is not possible to assess confounding factors that may be responsible for the improvement. While causation cannot be established, improvements were temporally related to the adoption of the recovery protocol, as the improvements in symptoms occurred rapidly after the dietary change and have been sustained, up to 7 years in one case, with adherence to the diet. Cases were not counseled with the goal of participating in scientific research, thus food diaries were not collected, and a nutrient analysis of intake was not conducted, nor has a nutrient analysis been conducted with respect to the protocol overall. Adherence was assessed via self-report only. In addition, dietary intake was self-reported by patients after completing the protocol and experiencing symptom remission. Future research should assess dietary change and changes in symptoms prospectively. Finally, the three cases presented reported that the dietary change was low burden relative to their debilitating symptoms and poor quality of life and, as such, they were highly motivated to adhere to the recovery protocol. This may not be the case for all patients, and adherence may be difficult for others attempting this diet. It should be noted that while Case 1 experienced great success with the protocol, pregnant individuals should be encouraged to consult with their obstetrician and medical team before implementing significant dietary changes.

In the United States, poor diet quality, including added sugar intake above recommendations, is a concern (64–67). The recovery protocol described shares similarities with a WFPB diet, particularly in its elimination of all animal products and processed foods. Theoretically, WFPB diets are more nutrient dense than the standard American diet (68), and although a WFPB diet may require supplementation of select nutrients, such as calcium, vitamin D, and vitamin B12 (68–70), a supplemented WFPB diet can be nutritionally sufficient and is less likely to be deficient in underconsumed nutrients such as fiber, potassium, Vitamins A, E, and C, and magnesium (71). Additionally, a WFPB diet does not provide an excess of the nutrients consumed in excess in the US, including saturated fat, sodium, and added sugar. With respect to calcium, plant foods such as leafy greens and broccoli are rich sources (72). The three cases described were advised to take vitamin B12 and D supplements; however, blood work was not conducted to examine nutritional deficiencies. With respect to the high amount of water consumption, it should be noted that all patients on the recovery protocol, including the three documented in this case series, are advised to consume at least 1 tsp. of sodium per day and remain under the care and monitoring of their own physician. Case 3 struggled with hyponatremia both before and over a year after completing the recovery protocol, for which she was carefully monitored and treated by her physician and still reported remarkable improvements in symptoms and quality of life following the dietary change.

In conclusion, three women reported remission of SLE and SS symptoms following adherence to a predominately raw, plant-based recovery protocol diet rich in leafy greens, cruciferous vegetables, and omega-3 fatty acids and eliminating all processed foods. Future research should examine the potential of the recovery protocol to reverse symptoms of SS and SLE in other patient populations. In addition, while this case series focuses on SLE and SS, it is possible that the recovery protocol may be successful in improving symptoms of other chronic diseases, and future research should eventually explore this possibility. A previous case series has documented similar results among patients with SLE-related nephritis (18), and clinicians may consider recommending dietary changes, in combination with pharmacological treatment, as a low-risk strategy with positive side effects for further ameliorating symptoms of autoimmune disease. It would be beneficial for fellow clinicians to document their successes managing autoimmune disease with dietary interventions as case reports. In light of the limited research on diet as a treatment for autoimmune disease, documented cases showing the possibility of lifestyle change as an avenue for treatment could eventually lead to larger-scale studies and, in the longer term, randomized controlled trials. Most importantly, practitioners and patients deserve to be made aware of the promising potential of using food as medicine in the treatment of SLE and SS.

## Data availability statement

The original contributions presented in the study are included in the article/Supplementary material, further inquiries can be directed to the corresponding author.

## Ethics statement

Ethical approval was not required for the studies involving humans because this was a case series of 3 cases. Written informed consent was obtained. The studies were conducted in accordance with the local legislation and institutional requirements. The participants provided their written informed consent to participate in this study. Written informed consent was obtained from the individual(s) for the publication of any potentially identifiable images or data included in this article. Written informed consent was obtained from the participant/patient(s) for the publication of this case report.

## Author contributions

BG is responsible for the conception and design of this work, developed the nutrition recovery protocol, directly oversaw the 3 patients described, and critically reviewed the manuscript. KS interviewed the patients and authored the manuscript. All authors contributed to the article and approved the submitted version.
