# Supplementary material for: Case series: raw, whole, plant-based nutrition protocol rapidly reverses symptoms in three women with systemic lupus erythematosus and Sjögren’s syndrome
Source: Front Nutr. 2024 Feb 27;11:1208074. doi: 10.3389/fnut.2024.1208074 (PMC10949923; doi:10.3389/fnut.2024.1208074)
Supplement: Supplementary file 1 [file Presentation_1.pdf]

Personal Testimonies

Case 1

By the time I discovered this protocol [recovery protocol], I already had one child who was 3 years old, I had one miscarriage in the second trimester due to “complications from lupus,” and I was 9 months pregnant with my second child. I decided I had no more time to waste and went in 100% on the protocol. I was shocked at how quickly I started feeling better while I was pregnant. My joint pains were gone within weeks. I noticed my chronic dry mouth and dry eyes were gone before 4 weeks. I remember being so excited when I finally started producing my own saliva for the first time in a couple of years! It took a couple of months for all the cracks to heal on my skin which some I had for over 6 years. The most exciting thing for me was when I realized being in direct sunlight didn’t hurt my skin. I’ll never forget the feeling of going to the beach 2 months after giving birth and enjoying the feeling of the warmth of the sun on my face and body. Prior to that, being in direct sunlight would physically hurt my skin. Now I can enjoy it! I remember realizing the possibilities were endless with what I could do for myself and what I could do with and for my family now that I was healthy.

My life has changed so much since doing this protocol, and I am forever grateful. I am now spending my days exercising, working full time, and I have energy at the end of the day to be active with my children. I spend so much time outside now. I recently [in 2020] had my third baby which I was told would be dangerous and almost impossible. It’s amazing to have my health, and I will never take it for granted. On top of that, I now have the tools to make sure my children all have a healthy start to their lives.

Case 2

I was first diagnosed with lupus then Sjögren’s syndrome. I had an itchy scalp, my skin was itching, my feet were itchy. I thought I had carpal tunnel syndrome. My wrist hurt all the time. I wore braces. I didn't even text [on her phone]. I was exhausted all the time. I had brain fog all the time. I actually started to think that I had early onset Alzheimer's disease. I would teach [for her job], and my ankles would hurt so much that I started sitting down when I would lecture to a class. I was buying vitamins. I was doing everything, and nothing was helping. I cannot emphasize how much it [the recovery protocol] changed my life. Immediately, the itching stopped. I don't have to wear braces on my wrist. I can type. I apparently don't have carpal tunnel syndrome. I don't have any ankle pain. I don't have any knee pain. I had one doctor that I had consulted [prior to the recovery protocol] say that I had a labral tear in my hip, and one option was hip surgery. I don't have any pain in my hip now. I can walk everywhere. I can play with my kids. I can remember things, and I don't have to write everything down. I can type, I can text, and I don't take any painkillers anymore. I had stomach problems for years; that is completely gone. My stomach is perfect. I can stand up in front of my class. I just have energy. It [the recovery protocol] has transformed every aspect of my life and the way I feel, and I hope that everybody who's suffering from any kind of autoimmune or other disorder has the opportunity to try it.

Case 3

I am a 45-year-old mother of four beautiful children and a teacher of students with special needs, and I used to have lupus and Sjögren’s syndrome. My symptoms began after I started having children in the early 2000’s. At the time, I was home with my children, and it was all very hard. I had severe brain fog, debilitating fatigue, nerve pain, Raynaud’s, joint pain, migraines that lasted for months, eye pain, dizziness, atypical trigeminal neuralgia, grittiness in my eyes, patches of skin that would hurt like they were sunburned, dry mouth that could never feel relieved, achiness, nausea, and a feeling of generally being unwell. I couldn’t exercise strenuously without feeling sick for days. I was diagnosed with Sjögren’s after my fourth child was born with neonatal lupus, which is not actually lupus, but is a rare occurrence when the infant’s body reacts to autoantibodies from the mother passed through the placenta. I was diagnosed with lupus several years later. In 2020, I got really sick from my autoimmune diseases. I discovered Dr. Goldner’s book through my mom, who had reversed her own fibromyalgia and had been on a magazine cover twice at age 70 after losing 100 pounds using a whole-food, plant-exclusive diet. I did the Rapid Recovery Program (RRP) with Dr. Goldner in 2021, and my lupus and Sjögren’s symptoms went away within weeks. I was able to stay up late. I wasn’t tired anymore. My skin and joint pain vanished. I didn’t feel nauseous anymore. My body felt amazing. My eye pain and grittiness went away, my migraines left, and my trigeminal neuralgia took a little longer to leave but eventually was gone. I stayed on the diet until my lab work fully normalized, a year after the RRP. Today, I continue to eat at least 2 lb of raw cruciferous veggies a day, high omega3’s, and follow a fully plant-exclusive diet. I have ZERO symptoms and feel AMAZING!! It is so good to feel well. I can exercise strenuously without difficulty! I am not tired anymore! No pain, no brain fog. I recently got my master's degree and can be a mom and also support my students effectively and without difficulty. This program has changed my life, and I can do I EVERYTHING I want to do without limitations! It absolutely feels amazing to be well!
